# NetGrep: fast network schema searches in interactomes

**DOI:** 10.1186/gb-2008-9-9-r138

**Published:** 2008-09-18

**Authors:** Eric Banks, Elena Nabieva, Ryan Peterson, Mona Singh

**Affiliations:** 1 Department of Computer Science, Princeton University, 35 Olden Street, Princeton, NJ 08540, USA; 2 Lewis-Sigler Institute for Integrative Genomics, Princeton University, Carl Icahn Lab, Princeton, NJ 08544, USA; 3 Current address: Department of Computer Science, Cornell University, 4130 Upson Hall, Ithaca, NY 14853, USA

## Abstract

NetGrep () is a system for searching protein interaction networks for matches to user-supplied 'network schemas'. Each schema consists of descriptions of proteins (for example, their molecular functions or putative domains) along with the desired topology and types of interactions among them. Schemas can thus describe domain-domain interactions, signaling and regulatory pathways, or more complex network patterns. NetGrep provides an advanced graphical interface for specifying schemas and fast algorithms for extracting their matches.

## Abstract 2

NetGrep is a system for searching protein interaction networks for matches to user-supplied network schemas.

## Rationale

High-throughput experimental and computational approaches to characterize proteins and their interactions have resulted in large-scale biological networks for many organisms. These complex networks are composed of a number of distinct types of interactions: these include interactions between proteins that interact physically, that participate in a synthetic lethal or epistatic relationship, that are coexpressed, or where one phosphorylates or regulates another (for a review, see [1]). Though incomplete and noisy, these networks provide a holistic view of the functioning of the cell, and with appropriate computational analysis and experimental work have significant potential for helping to uncover precisely how complex biological processes are accomplished.

We have developed a network analysis system based on querying interactomes using templates corresponding to network patterns of interest. Searching for recurring patterns in biological data has been the backbone of much research in computational biology; for example, within the context of sequence analysis, it has given rise to extensive work on sequence alignments and sequence motif discovery and has resulted in large sequence motif libraries. Not surprisingly, within the burgeoning field of biological network analysis, considerable effort has been focused on uncovering recurring patterns within interactomes. Mapping homologous proteins with conserved interaction patterns in different interactomes has revealed shared modules and complexes recurring across a range of organisms [2-6]. Analysis of the wiring diagrams of interactomes has uncovered network motifs that occur more frequently than expected by chance [7-13]. Additionally, there has been much work on uncovering recurring domain-domain interactions in physical interactomes [14-23], both to suggest a physical basis for known interactions and to help predict new interactions. Most closely related to the work described here are previous attempts to query biological networks using particular user-supplied subgraphs [24-29].

**Figure 1:**
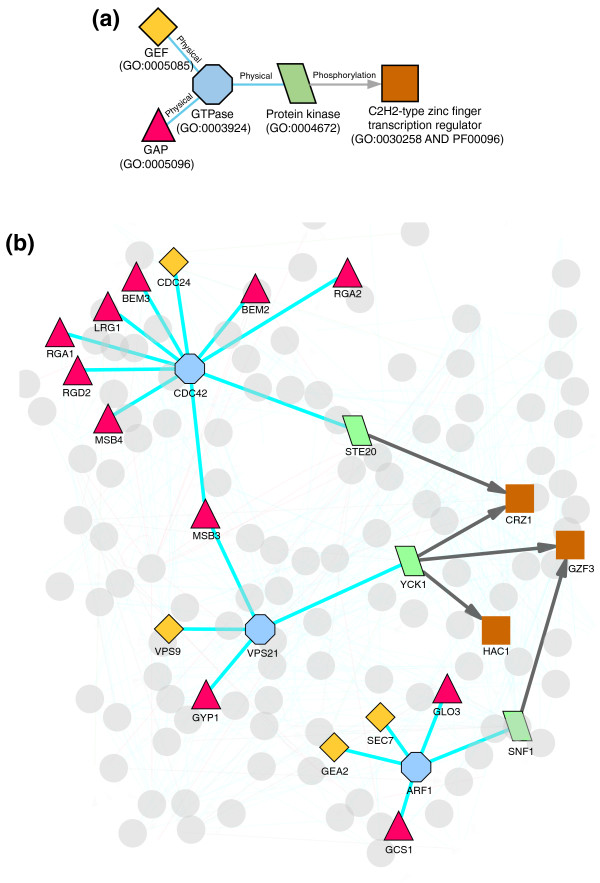
A sample schema and its instances in yeast. **(a) **An example of a schema. Each protein in the schema has a specific feature description and each edge has a type. In this case, the schema describes Ras GTPase signaling, where small G proteins from the Ras family are regulated by GTPase activating proteins (GAPs) and Guanine nucleotide exchange factors (GEFs), and in turn regulate effector kinases, which may phosphorylate other proteins. **(b) **Instances of the schema in *S. cerevisiae*.

**Figure 2:**
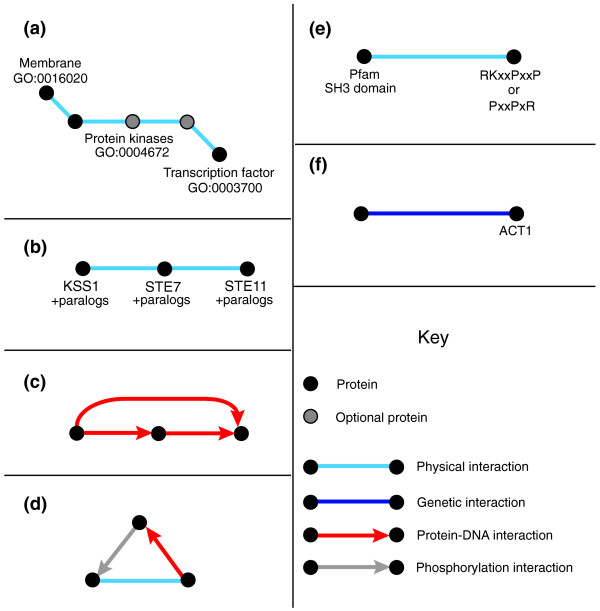
Sample schemas. Examples of network schemas. Unlabeled schema proteins are considered to be 'wildcards' and can match any protein in the interaction network. **(a) **A signaling pathway schema. This schema matches all sets of proteins such that a protein in the cell membrane physically interacts with a succession of anywhere between one and three kinases, the last of which physically interacts with a protein that is a transcription factor. **(b) **A MAP kinase schema, specified by particular yeast proteins making up a canonical MAPK signaling pathway. **(c) **A feed-forward loop network motif [8] schema. The unlabeled nodes can match any protein in the network. **(d) **A 'kinate' feedback loop network motif schema [13]. **(e) **An SH3 domain interaction schema. This schema matches all interacting pairs of proteins such that one contains a Pfam SH3 domain and the other has one of the specified patterns, corresponding to SH3 binding sites, in its underlying amino acid sequence. Amino acids in the pattern are specified by their one letter code, and 'x' denotes a match to any amino acid. **(f) **A specific protein schema. This schema matches all proteins with a synthetic lethal relationship to yeast protein ACT1.

In this paper, we introduce a system, NetGrep, that integrates the wealth of prior information known about individual proteins - for example, their functional annotations, sequence motifs, predicted domain structures, or other attributes - within the context of user-directed network searches. In particular, NetGrep utilizes 'network schemas' to describe patterns in interaction networks and incorporates fast algorithms to search for matches of these schemas within networks. A network schema describes a group of proteins with specific characteristics and with the desired topology and types of interactions connecting them (Figure 1a). A schema's matches, or instances, in an interactome are subgraphs of the interaction network that are made up of proteins having the specified characteristics, which interact with one another as dictated by the schema's topology (Figure 1b). In graph-theoretic terms, a schema corresponds to a graph with labeled nodes and edges, and finding instances of a schema within an interactome corresponds to solving a subgraph isomorphism problem. The NetGrep system allows querying with schemas described via a diverse set of protein features, including Prosite family [30], Pfam motif [31], SMART domain [32,33], Supfam superfamily [34], and Gene Ontology (GO) [35] annotations. Proteins may also be specified via particular protein IDs, homology to other proteins, regular expressions over amino acids, or with unions or intersections over any of the previously described features. By utilizing these protein attributes in combination with physical, genetic, phosphorylation, regulatory, and/or coexpression interactions (as available for the organism of interest via high-throughput experiments), the network schema queries allowed in NetGrep generalize many previously studied interaction patterns. For example, a general network schema relating to signaling is a path of physically interacting proteins, where the first protein is a receptor, and the last protein is a transcription factor (Figure 2a); such queries have been used in conjunction with gene expression data to infer signaling pathways in *Saccharomyces cerevisiae *[36]. A more specific network schema relating to signaling consists of particular proteins making up a pathway that can be used to search for paralogous pathways (Figure 2b), as has been suggested in network alignment approaches [37]. Network motifs have been widely studied [7,8], and can be described by schemas without constraints on protein types but with particular interaction types specified (Figures 2c,d). Domain-domain or domain-peptide interactions, such as those important for cell signaling and regulatory systems [38], can be represented by two-protein schemas with the proteins appropriately constrained (Figure 2e). Schemas relating to specific proteins of interest are also easily incorporated (Figure 2f). Finally, network schemas can be naturally extended to handle approximate matches by specifying optional nodes (Figure 2a). While these types of network interaction patterns have been studied in a wide-range of contexts, it has not even been possible to use many of them as queries in existing systems. Thus, we have introduced NetGrep to provide a flexible, unified system for interrogating an interactome using a diverse set of queries.

In addition to allowing a broad range of network schema queries, NetGrep has an easy-to-use graphical interface for inputting schemas. For each user-input schema, NetGrep finds all of its matches in the chosen interactome. Although the search problem is a case of the computationally difficult subgraph isomorphism problem, we have been able to develop algorithms that take advantage of schema characteristics for biological networks. As a result, NetGrep's core algorithms are extremely fast in practice for queries with up to several thousand matches in the interactomes studied. Though speed is useful for individual user queries, it also makes it possible to systematically enumerate and query many network interaction patterns. For example, here we have systematically tested NetGrep's underlying algorithms by enumerating >100,000 schema queries with proteins described via GO molecular function terms and have found that for schemas with up to tens of thousands of matches, NetGrep can rapidly uncover all instances. Our algorithms can thus enable new analysis that characterizes networks with respect to the types and numbers of interaction patterns found (for example, see [39]).

## Relationship to previous work

There are several previously developed tools for querying biological networks. While none of them have the functionality of NetGrep, we briefly review them here. Previous approaches fall broadly into the categories of network alignment, network motif finding, and specific subgraph queries, although these categories overlap.

Network alignment tools [4,5,37,40] align protein-protein interaction networks by combining interaction topology and protein sequence similarity to identify conserved pathways. These tools can be used to identify schemas for which the criterion for matching a query protein to a target protein is sequence similarity. Network alignment has also been applied to metabolic networks [24], with proteins characterized by their enzyme classification. Algorithmically, these approaches are designed for aligning entire interactomes, and several of them are based on local alignments based on simpler linear or tree topologies. NetGrep in contrast is developed and optimized for general network schema queries, and has faster algorithms for the task at hand.

Several tools exist for uncovering network motifs or over-represented topological patterns in graphs [41,42], and these could be used to find schemas consisting solely of unannotated proteins. These approaches do not, however, provide a mechanism for utilizing specific protein annotations, nor do they allow user defined queries. We note that while NetGrep can obtain instances to network motif queries, our algorithms are optimized for schemas utilizing protein descriptions and with up to tens of thousands of instances. Alternative algorithms, specifically developed for counting or approximating the total number of instances of network motifs [43,44], may be more suitable if network motif queries are desired.

Other more closely related tools have been implemented to query biological networks using subgraphs. Given a linear sequence of GO functional attributes, Narada [45] finds all occurrences of the corresponding linear paths in a network. MOTUS [25] is designed for non-topology constrained subgraph searches in metabolic networks. Qnet [28] is restricted to tree queries and utilizes only sequence similarity. NetMatch [26], extending ideas of GraphGrep [46], allows users to search for subgraphs within the Cytoscape [47] environment and can be used for simple schema queries. SAGA [27] is a subgraph matching tool for Linux platforms that allows inexact matches to a query in multiple networks, and has built-in support for biological networks where proteins are described via orthologous groups. In contrast to these approaches, NetGrep is a standalone, multi-platform system where schemas may have arbitrary topologies as well as a large set of built-in protein and interaction types. NetGrep schemas allow flexibility via optional nodes (thereby permitting inexact matches) and protein and interaction descriptions that may consist of boolean conjunctions or disjunctions of features. While NetGrep comes with built-in protein feature and interaction data sets for several model organisms, it also has the ability to incorporate new custom networks and associated feature sets. Furthermore, NetGrep can optionally be used within the Cytoscape environment to visualize schema matches. See Table 1 for a comparison of features available in NetGrep and previous approaches.

**Table 1:** Feature comparisons

Feature	PathBLAST [37]	Fanmod [41]	Narada [45]	SAGA [27]	NetMatch [26]	NetGrep
Non-linear queries	X	X		X	X	X
Allows arbitrary protein annotations		1 per node			Unlimited	Unlimited
Boolean combination of annotations				X		X
Inexact matches	X			X		X
Multiple edge types in a network		X			X	X
Boolean combination of edge types						X
UI for searching/choosing annotations			X			X
Can be used with Cytoscape					X	X
Can be used as a standalone	X	X	X	X		X
Custom data sets provided	X			X		X

A comparison of built-in features available in systems that can, in principle, be used for querying interactomes using network schemas. A network alignment tool, PathBLAST, and a network motif finder, Fanmod, are shown for comparison. All other systems are explicitly designed for querying interactomes utilizing labeled subgraphs.

## Implementation

We have implemented NetGrep in Java so that it is easily portable among different operating systems. Users have the option of running a feature-limited version of the software on our server [48] or of downloading the fully featured program and running it locally. NetGrep can be used both as a standalone application or in conjunction with Cytoscape as a plugin if visualization of the results in network form is desired. A detailed description of how to use NetGrep is provided online [49]. More formal descriptions of schemas, their instances in the interactome, and the algorithms used to uncover the instances are given in the 'Model and algorithm' section below.

### Packaged data files

Data files are provided for the following model organisms to be used with NetGrep: *S. cerevisiae*, *Caenorhabditis elegans*, *Drosophila melanogaster *and *Homo sapiens*. These files contain all the information necessary to run NetGrep, including protein information (names and aliases), interaction maps, and protein features.

Tables 2 and 3 list the protein features and edge types included in these data files. Physical and genetic interactions for all organisms are obtained from BioGrid [50] (version 2.0.34), and phosphorylation interactions for yeast are obtained from [13]. Regulatory relationships in yeast are obtained from the binding data of [51] using a *p*-value/cutoff of 1e-5. Gene expression interactions between pairs of proteins are taken as those that have linear correlation coefficient >0.8 on the concatenation of all experiments in the gene coexpression data compiled by [52]; we note that this high cutoff and required correlation in all conditions favors expression interactions between housekeeping proteins.

**Table 2:** Protein features

Protein feature	Source
Gene names and aliases	BioGRID [50] 2.0.34
Amino acid sequences	Biomart [58]
Paralogs	COG [59], Biomart [58]
Pfam A/B motifs	Pfam [31] 21.0
SMART [32,33] motifs	InterPro [60] 15.0
Prosite [30] motifs	
SCOP [34] superfamilies	
GO functional annotations	GO [35] 05/2007 download

Protein features used to annotate proteins in the built-in data sets provided with NetGrep.

**Table 3:** Interaction types

Interaction type	Source	Restrictions
Physical	BioGRID [50] 2.0.34	
Genetic		
Gene coexpression	[52]	
Transcriptional regulation	[51]	Yeast only
Phosphorylation	[13]	Yeast only

One important feature of NetGrep is that none of the data are hard-coded into the program. Users can therefore use any node features or edge types desired when constructing networks; for example, custom or newly defined interaction types can be added. Additionally, creating data files for other, non-supported organisms is a straightforward process.

### Describing proteins and interactions

**Figure 3:**
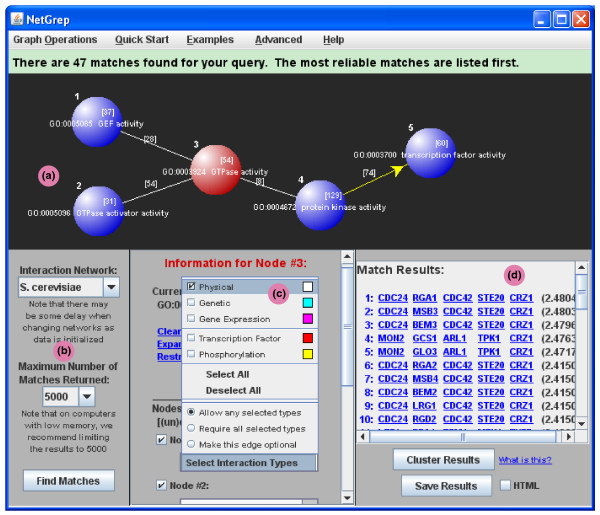
NetGrep screenshot. A detailed screenshot of the NetGrep display showing a sample query schema. **(a) **The graph panel area used to describe schemas. The Ras GTPase signaling schema from Figure 1 is shown in the panel with the Ras GTPase node highlighted. **(b) **The panel used to designate which interaction network to use, to choose the maximum number of matches desired, and to initiate a search. **(c) **The panel used to annotate nodes in the schema and to create or modify edges. The information for the highlighted node (node 3) is currently displayed in the panel; the edge between the first and third nodes is being modified. **(d) **The results panel in which the matches found from the search are displayed. Each row lists the proteins that make up a particular match along with its reliability score.

Nodes, describing proteins, are added to a schema via a visual canvas, and then individual features of the proteins can be selected (Figure 3a). The interactome to be queried is specified via a pull-down menu (Figure 3b). Each of the nodes in a schema can be annotated with any combination of protein features; multiple features are related by boolean combinations via ANDs or ORs. A node in a schema can be connected to any other, corresponding to a desired interaction, also by specifying this in the visual canvas. These edges between nodes can be described as having one or more types (Figure 3c). As with protein features, edge types may be combined with logical ANDs or ORs. For example, one might require that two given proteins physically interact AND that the first is a transcription factor regulating the second. Note that a schema must be a connected graph.

### Specifying inexact matches

The schemas described thus far are rigid in their structure. Occasionally, a user might prefer to specify that any number of proteins with a particular feature set interact in a cascade or that a given node in the schema not be absolutely required. NetGrep achieves this flexibility by allowing nodes in the schema to be designated as optional. When a schema contains an optional node, NetGrep will find matches both with and without the given protein. For example, to represent a signaling pathway as 'a protein in the membrane, which interacts with a succession of between one and three kinases, the last of which interacts with a transcription factor', one would build the given linear five-node pathway and designate two of the kinases as optional (Figure 2a). NetGrep would then find all three-, four-, and five-node matches within the network. Note that single nodes with more than two interactions cannot be designated as optional. When an optional node has two interactions, the interaction types are logically ORed for instances of the schema that have the optional node excluded.

Similarly, a significant problem with current interaction datasets is that they are incomplete. NetGrep provides a solution to this difficulty by also allowing interactions in a schema to be designated as optional. When a schema contains an optional interaction, NetGrep will allow matches even if the given interaction is not found in the network.

### Matches and reliabilities

NetGrep has a user-set threshold that limits the number of matches reported for an input schema (Figure 3b). As a typical user is not expected to look through tens of thousands of matches, this threshold can be as low as 100 and as high as 50,000. For faster run times, a lower threshold is recommended; additionally, the threshold limits memory usage. Alternatively, if the total number of instances is greater than the highest allowed threshold, there is an advanced (somewhat slower) option that computes the total number of instances but does not explicitly enumerate them.

The instances of a query schema are returned by NetGrep, up to the user-defined threshold, and are sorted according to how confident we are of the underlying interactions. In particular, for each pair of proteins, we have a single precomputed reliability value between 0 and 1 that assesses how likely these two proteins are to interact (see 'Interaction reliabilities' section below). For each of the matches found by NetGrep, its overall reliability is computed by multiplying together the reliabilities corresponding to protein pairs that have interactions in the matches. The matches are sorted based on the negative log of this value, beginning with the most reliable (Figure 3d).

## Performance

We have found NetGrep to run extremely fast in practice. We illustrate the performance of NetGrep in two ways. First, we report how long NetGrep takes for each of the schemas shown in Figure 2. As a comparison, whenever possible, we have also run these schemas on the same network using other tools. For each system, the software is downloaded and run on a laptop running Windows XP with 1 GB RAM and a 1.66 GHz Intel processor. All queries are run on our *S. cerevisiae *network data, described above. All timings include the times for both the search and output of the results. Default settings for all programs are used. While we have NetGrep print out its wall clock time to standard output, the timings for the other systems are estimated via a handheld timer and rounded down to the nearest second. We have chosen this process as some of the systems must be run within a graphical interface and strict system timing calls are not possible. Each query is repeated ten times and the reported running times are the averages over these runs. Table 4 shows the performances for each sample query. Note that table entries are left blank for schemas that cannot be run on a given system and two of these queries can currently be run only on NetGrep. NetGrep has considerably faster query times for all sample queries, and is often more than an order of magnitude faster than previous approaches.

**Table 4:** Running time comparisons

	Running time (s)				
	
Sample query	PathBLAST	Fanmod	Narada	NetMatch	NetGrep
Signaling pathway 1			28		4.2
Signaling pathway 2					26.9
MAPK pathway	90				0.02
Feed-forward motif		32		5.2	1.4
Kinate motif		32		5	0.5
SH3 domain interaction					0.5
ACT1 genetic interaction				15	0.1

Running times (in seconds) for several sample queries on the *S. cerevisiae *interaction network, using PathBLAST, Fanmod, Narada, NetMatch and NetGrep. All reported running times are for search and output only. As in Table 1, PathBLAST is used as a prototypical example of a network alignment tool and Fanmod represents network motif finders. Note that SAGA is excluded here because it cannot be run on Windows. The sample schemas correspond to those provided in Figure 2, except that two distinct queries are used for Figure 2a. In the first, all three kinases in the pathway are required. In the second, two of the kinases are designated as optional (as in Figure 2a). Each query is run ten times and the average computation time is provided. Row entries are left blank for any tool that is unable to find instances of a particular schema because of feature limitations.

**Figure 4:**
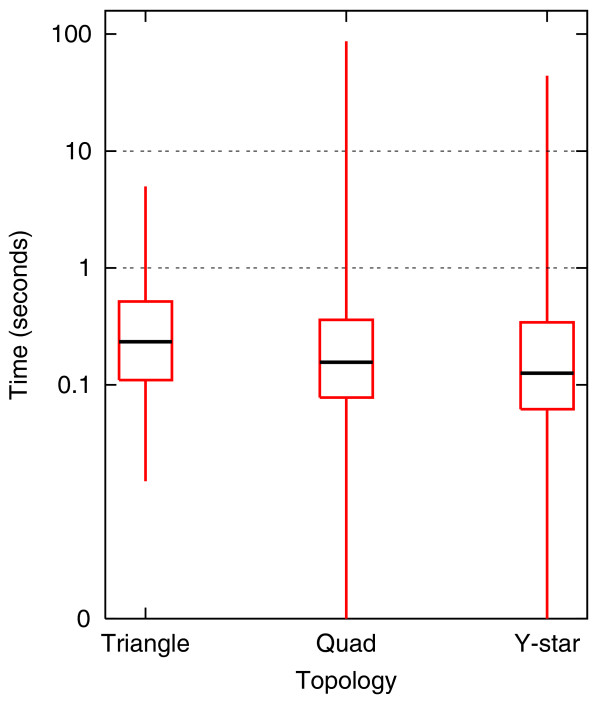
Yeast GO molecular function schema timings. All possible triangular, 4-node linear, and 4-node branched schemas ('Y-star') with nodes described via GO molecular function slim terms were run systematically on NetGrep. Results are reported for those schemas with at least 5 but no more than 80,000 instances in *S. cerevisiae*: 780 triangular schemas; 80,719 4-node linear schemas; and 30,642 4-node branched schemas. Boxplots of the running times for each topology are given; boxplots are a convenient way of depicting the smallest observation, second quartile, median, third quartile, and largest observation in the data.

Second, we have run NetGrep in a systematic fashion on schemas consisting of physical interactions in triangular, 4-node linear 'quad,' and 4-node branched (that is, a central node interacting with three others) 'Y-star' topologies. We consider all possible ways to annotate the proteins in these topologies using GO molecular function slim [53] terms (see Additional data file 1 for terms used). We have chosen these types of schemas because of their linear, branched, and cyclical topologies, and because we are easily able to exhaustively enumerate over all possible schemas of this type on a standard laptop. Additionally, GO annotations can be utilized with queries in two previous systems, NetMatch and Narada (though Narada is limited to the linear schemas). There are 1,771 triangular schemas, 101,871 quad schemas, and 37,191 Y-star GO molecular function slim schemas. Since each GO slim term is general and can annotate many proteins, we set the threshold for the maximum number of matches allowed to 80,000. Of the schemas, almost all have fewer than 80,000 instances in *S. cerevisiae *(all triangular schemas, 97,170 quad schemas and 37,129 Y-star schemas). Statistics about how long NetGrep takes to retrieve all instances for each query that has between 5 and 80,000 instances in yeast are given in Figure 4; we exclude schemas with fewer than 5 matches as they typically take less time. As can be seen, matches for each of these queries are found within 100 seconds, but the vast majority in fact take less than even 10 seconds. We are not able to time NetMatch and Narada in a systematic manner; thus, we have arbitrarily chosen three triangle, five quad, and five Y-star molecular function queries, to give a sampling of run times for these previous approaches on these types of schemas. The schemas and their timings are shown in Table 5.

**Table 5:** GO MF running time comparisons

		Running time (s)		
		
Topology	Query	Narada	NetMatch	NetGrep
Triangle	GO:0003677, GO:0004386, GO:0004672		15	0.1
Triangle	GO:0004386, GO:0004672, GO:0030528		16	0.2
Triangle	GO:0003723, GO:0003723, GO:0003723		15	1.9
Quad	GO:0004386, GO:0003677, GO:0016874, GO:0016829	1	14	0.2
Quad	GO:0016787, GO:0030234, GO:0005515, GO:0008233	2.3	17	1.2
Quad	GO:0003677, GO:0003723, GO:0005515, GO:0005198	4	16	1.9
Quad	GO:0016787, GO:0005198, GO:0003677, GO:0016779	2.2	17	1.7
Quad	GO:0016787, GO:0016740, GO:0016779, GO:0030528	4.8	16	2.9
Y-star	GO:0008233, GO:0016874, GO:0030234, GO:0005215		15	0.2
Y-star	GO:0005515, GO:0004721, GO:0008233, GO:0016740		17	0.8
Y-star	GO:0005515, GO:0008233, GO:0005198, GO:0005215		17	3.9
Y-star	GO:0030528, GO:0005515, GO:0016740, GO:0005215		14	1.5
Y-star	GO:0016740, GO:0005515, GO:0030528, GO:0005215		14	5.2

A comparison of running times (in seconds) for several sample schemas annotated with GO molecular function slim terms on the *S. cerevisiae *interaction network using Narada, NetMatch and NetGrep. Of the previous methods, Narada and NetMatch are chosen as they can be run off-the-shelf for these schemas; note, however, that Narada only handles linear topology queries. All reported running times are for search and output only. In the case of the Y-stars, the first term shown annotates the central node. The schemas shown have between 10 and 11,000 instances in *S. cerevisiae*.

## Model and algorithm

### Graph model

We give a formal specification of the problem. Let *L *be the set of possible protein labels (for example, Pfam motifs, protein IDs, and so on) and let *T *be the set of possible edge types (for example, physical, regulatory, and so on). An interaction network is represented as a mixed graph *G *= (*V*_*N*_,*E*_*N*_,*A*_*N*_). *V*_*N *_ is the set of *vertices*, with a vertex *v *∈ *V*_*N *_ for each protein. *E*_*N *_⊆ *V*_*N *_× *V*_*N  *_is the set of undirected edges, and *A*_*N *_⊆ *V*_*N *_× *V*_*N  *_is the set of arcs or directed edges. Vertices correspond to proteins and edges and arcs correspond to interactions. Each vertex *v *in the interaction network is associated with a set of features *l*(*v*) ⊂ *L *(specifying protein features), each edge (*u*,*v*) is associated with a set of types *t*_*e*_(*u*,*v*) ∈ *T *(specifying the undirected interactions between the proteins), and each arc (*u*,*v*) is associated with a set of types *t*_*a*_(*u*,*v*) ∈ *T *(specifying the directed interactions between the proteins). If there is no edge between *u *and *v*, *t*_*e*_(*u*,*v*) = ∅, and if there is no arc between *u *and *v*, *t*_*a*_(*u*,*v*) = ∅.

A network schema is a mixed graph *H *= (*V*_*S*_,*E*_*S*_,*A*_*S*_) such that: (1) each vertex *v *∈ *V*_*S *_is associated with description set *D*_*v *_such that each *d *∈ *D*_*v *_is a subset of *L *(in NetGrep, the set *D*_*v *_is constructed via individual protein features in *L *and utilizing either intersections or unions over these features; for example, for a particular vertex *v *∈ *V*_*S*_, if a union is taken over individual feature types, *D*_*v *_consists of singleton sets consisting of each of these features; note that *D*_*v *_can consist of one set, the emptyset, in the case of a wildcard vertex); (2) for every pair of vertices *u *and *v *such that (*u*,*v*) ∈ *E*_*S *_∪ *A*_*S*_, there is an associated description set *D*'_*u*,*v *_⊂ *T *(in NetGrep, the set *D*'_*u*,*v *_ is constructed via individual interaction types, and requiring either all of them, or just one of them; for example, for a particular pair of vertices *u *and *v *with desired edges or arcs between them, if all interactions are required, then *D*'_*u*,*v *_consists of a single set consisting of all desired interaction types).

An instance of a network schema *H *in an interaction network *G *(that is, a match in the network for the schema) is a subgraph (*V*_*I*_,*E*_*I*_,*A*_*I*_) where *V*_*I *_⊂ *V*_*N*_, *E*_*I *_⊂ *E*_*N*_, and *A*_*I *_⊂ *A*_*N *_such that there is a one-to-one mapping *f*:*V*_*S*_→*V*_*I  *_where: (1) for each *v *∈ *V*_*S*_, there exists a *d *∈ *D*_*v *_such that *d *⊂ *l*(*f*(*v*)); (2) for each pair of vertices *u*,*v *∈ *V*_*S *_with (*u*,*v*) ∈ *E*_*S *_∪ *A*_*S*_, there exists a *d*' ∈ *D*'_*u*,*v *_such that *d*' ⊂ (*t*_*e*_(*f*(*u*),*f*(*v*)) ∪ *t*_*a*_(*f*(*u*),*f*(*v*))). Note that two distinct instances of a schema may share proteins and/or interactions; however, any two instances must differ in at least one protein. Network schemas are used to interrogate the interaction network for sets of proteins that match this description.

### Interaction reliability

For each pair of proteins, we estimate the reliability of their having any interaction between them. In particular, we first partition all the observed underlying interactions in the interactome into several experimental groups. The reliability of each experimental group *i *is then evaluated as follows. For experiments determining non-genetic interactions, the reliability is estimated based on 'functional coherence' by computing *s*_*i *_as the fraction of interactions in that group that are between proteins sharing a high-level GO biological process slim term [53] (only pairs of interacting proteins that both have GO slim annotations are considered). We note that we do not use the functional coherence measure to assess genetic interaction experiments, as these types of interactions can bridge between pathways [54]. Instead, for these experiments, the reliability is estimated based on a '2-hop' topological measure that has been shown to be highly predictive of genetic interactions [55]. In particular, the reliability *s*_*i *_for an experimental group determining genetic interactions is estimated by computing the fraction of interactions in that group that additionally have paths of length two between them in the full interactome where either both interactions are genetic interactions or where one is a genetic interaction and the other is a physical interaction. Then, for a pair of proteins *u *and *v*, we consider all interactions *j *found between them, and treat them as independent events. The reliability *r*(*u*,*v*) between *u *and *v *is then computed as:

*r*(*u*,*v*) = 1 - Π_*j*_(1 - *s*_*g*(*j*)_)

where *j *ranges over all interactions linking proteins *u *and *v*, and *g*(*j*) gives the experimental group of interaction *j*. If no interactions exist between the two proteins, *r*(*u*,*v*) = 0. This noisy-or scheme is similar to the one used for reliability estimation in [56,57].

We partition our interactions into the following experimental groups. For physical and genetic interactions, there is one group for each individual high-throughput physical and genetic interaction experiment (defined as those that discover at least 50 interactions). All small-scale physical interaction experiments (defined as those that discover fewer than 50 interactions) are considered as belonging to a single group. Similarly, small-scale genetic interaction experiments are considered a single group. Experiments are identified by the combination of 'Experimental System' and 'PubMed ID' as reported by the BioGRID [50]. All phosphorylation interactions in [13] are considered in one group. In the case of interactions that are associated with continuous numerical data, such as coexpression interactions (associated with the correlation coefficient) and regulatory interactions [51] (associated with the *p*-value for the binding), we assign each interaction to one of 20 uniform bins associated with the numerical data, and consider each bin as a separate group.

### Searching for schemas

#### Overview

Finding the matches for a particular schema in a network corresponds to the computationally difficult subgraph isomorphism problem. A number of sophisticated algorithmic approaches for closely related problems on biological networks have been introduced earlier (for example, utilizing color coding [28]). Here, we obtain fast matches in practice utilizing a few key ideas. First, we pre-process the interactome to build fast look up tables mapping protein and interaction type labels to proteins associated with the labels. For each node in a schema, this allows us to quickly enumerate the set of all proteins that match the node's feature set. Second, we utilize the labeled schema nodes and schema edges to prune the search space. In particular, we constrain the proteins in each node match set by determining interaction matches along each edge in the schema. Finally, these interactions are cached for fast lookup in the last step, in which we enumerate the considerably smaller search space, and construct the full list of matches. We describe these steps in more detail below.

#### Algorithm

We first pre-process the interactome to maintain two hashes that map labels to proteins associated with those labels. *HASH*_*F *_maps protein features to sets of vertices described by those features (for example, all kinases), and *HASH*_*T *_maps edge types to pairs of proteins connected by an edge annotated with the types (for example, all proteins with physical interactions). For directed edge types, there are two separate entries in *HASH*_*T*_, one for each direction of the edge (for example, one for all kinases and one for all substrates). These hashes are used to quickly build, for any schema, its matches edge by edge.

When searching for instances of a particular schema, we associate with each node *v *in the schema a set of node matches *NMATCH*_*v*_, which contains all of the proteins in the interaction network that are described by that particular schema node (that is, the proteins that could be a match to that schema node). Specifically, we use *HASH*_*F *_to initialize *NMATCH*_*v *_with all the proteins that match *v*'s feature set. When features are combined with a boolean AND, we take the intersection of the protein sets from *HASH*_*F*_, and when they are combined with a boolean OR, we take the union of the protein sets. For each edge *e *= (*u*,*v*) in the schema that has a single type (that is, is not composed of a boolean combination of types) or for which all edge types are required (that is, types are combined by a logical AND), we use *HASH*_*T *_to trim the proteins in each node match set. For example, if schema node *v *is connected by a physical edge, then we can remove all proteins from *NMATCH*_*v *_that are not found in the set from *HASH*_*T *_corresponding to all proteins in the network connected by a physical edge.

We next prune the sets of node matches as follows, or until any of them becomes empty (at which point we know that there are no matches to the query in the network). For each edge *e *= (*u*,*v*) in the schema, we use the network interaction map to remove all proteins from *NMATCH*_*u *_that do not interact with any of the proteins in *NMATCH*_*v *_given *e*'s specified type. Although we could repeat this pruning step after each edge is processed, we have found it to be unnecessary because of two additional optimizations that we introduce. First, as we iterate through the edges in this step, we start with those edges whose endpoints contain the smallest sets of node matches and we progress in order; this optimization helps to reduce the size of the larger node match sets early on in the process. That is, we rank schema nodes based on the size of their node match sets, start with the node with the smallest node match set, and consider its edges first, starting with the neighbor with the smallest node match set. We then consider the node with the next smallest node match set, and so on. Second, as we iterate through the schema edges, we cache the matches for each edge, so that they can be quickly accessed in the next step where we find the actual matches. Note that this pruning step is skipped with optional nodes because edges connected to those nodes are not required. This pruning step is also skipped for edges if their match bins are too large (>1,000).

To find the sets of proteins that match the given schema, we iterate through each of the node match sets from smallest to largest, constructing matches as we go along. We note that this search order over the nodes provides a significant speed-up over a simpler approach that performs depth-first search from an arbitrary starting node in the schema. As we iterate through the nodes, for each protein *p *in a given match set representing node *v *in the schema, we constrain each larger match set representing node *u *in the schema as follows: if *u *and *v *are connected by an edge in the schema, we eliminate all proteins in *u*'s match set that do not interact with *p *(using the cached matches from the pruning step above). Furthermore, we remove *p *from *u*'s set if it is there (that is, we do not allow the same protein to occur in multiple positions of a match). We then set *p *as the matching protein at schema node *v *for this particular set of matches and traverse to the next largest node match set. Once a complete match to a schema is found, we backtrack and continue the search process.

If at any point the number of matches to a schema exceeds the user-defined threshold (Figure 2b), the search is terminated and NetGrep returns just those matches found up to that point. Once all matches to a schema are found, they are sorted by their interaction reliability, as described above.

#### Symmetric schemas

When a schema displays an inherent symmetry, it is often the case that the same set of proteins redundantly occurs in multiple instances. Consider, for example, the symmetric linear three-node schema *A*-*B*-*A*, where the edges are undirected, and the first and last nodes have identical feature sets and are symmetric around the middle node. One might find among the matches of this schema the proteins *p*_1_*-p*_2_*-p*_3 _and *p*_3_-*p*_2_-*p*_1_. NetGrep is able to determine that a given schema is symmetric and excludes these superfluous matches from the results returned by the search. The test for symmetry exploits the fact that for any two given nodes in a schema to be symmetric they need to have the exact same feature set and degree; for all pairs of nodes *u *and *v *in the schema for which this is true, the algorithm recursively checks all pairs of nodes connected to these two target nodes (that is, one connected to *u *and one connected to *v*) for symmetry, following any given edge just one time. This is equivalent to a depth first search over the schema. The base case in the recursive algorithm occurs when two target nodes are connected to each other or when they are connected to the same node.

If a query is determined to be symmetric, redundant matches are ignored during the search. To accomplish this task, each protein in the interaction network is first assigned an arbitrary unique ID number, as are each of the nodes in the query schema. Then, for any two symmetric nodes A and B in a query schema where the ID of A is smaller than the ID of B, we require that the ID of any protein matching node A be smaller than the ID of a protein matching node B in any given instance. All instances for which this requirement is not met for each of the symmetric nodes are ignored.

## Conclusion

We have introduced NetGrep, a powerful Java system for searching protein interactomes for instances of user-supplied labeled subgraphs, or network schemas, and have provided fully-featured data files for several organisms. NetGrep allows a wide-range of possible queries that supersede many previously studied interaction patterns. Finally, we have described an algorithm for solving the labeled subgraph isomorphism problem that is fast and effective in practice for biological networks.

## Availability and requirements

Project name: NetGrep

Project home page: [48]

Operating systems: Windows, Mac OS, Linux

Programming language: Java

Other requirements: Java 1.5 or higher

License: Open source with GNU General Public License

## Abbreviations

GO, Gene Ontology.

## Authors' contributions

EB designed and implemented the algorithms and system, performed data analysis, and co-wrote the paper. EN integrated interaction data sets, performed data analysis, and co-wrote the paper. RP developed software for the system. MS conceived and supervised the study, performed data analysis, and co-wrote the paper.

## Additional data files

The following additional data are available. Additional data file 1 is a table listing the GO molecular function slim terms used in the systematic testing of our program.

## Supplementary Material

Additional data file 1 GO molecular function slim terms used in the systematic testing of our program. Click here for file
