# Supplementary material for: NetGrep: fast network schema searches in interactomes
Source: Genome Biol. 2008 Sep 18;9(9):R138. doi: 10.1186/gb-2008-9-9-r138 (PMC2592716; doi:10.1186/gb-2008-9-9-r138)
Supplement: Additional data file 1 — GO molecular function slim terms used in the systematic testing of our program. [file gb-2008-9-9-r138-S1.pdf]

Additional file 1: GO molecular function terms utilized

GO ID	Description
GO:0003677	DNA binding
GO:0003723	RNA binding
GO:0003774	motor activity
GO:0004386	helicase activity
GO:0004672	protein kinase activity
GO:0004721	phosphoprotein phosphatase activity
GO:0004871	signal transducer activity
GO:0005198	structural molecule activity
GO:0005215	transporter activity
GO:0005515	protein binding
GO:0008233	peptidase activity
GO:0016491	oxidoreductase activity
GO:0016740	transferase activity
GO:0016779	nucleotidyltransferase activity
GO:0016787	hydrolase activity
GO:0016829	lyase activity
GO:0016853	isomerase activity
GO:0016874	ligase activity
GO:0030234	enzyme regulator activity
GO:0030528	transcription regulator activity
GO:0045182	translation regulator activity
